# Notes on Preparations for the Sick

**Published:** 1900-12

**Authors:** 


### Notes on Preparations for the Sick.

**Stypticin.**—According to Merck's *Digest* this preparation is a derivative of the opium alkaloid narcotine. It exists as a colourless powder, which is freely soluble in water. Chemically and physiologically it resembles hydrastine, in being an excellent uterine hæmostatic; but in addition it is said to possess sedative and analgesic properties. The dose is one grain, taken three to six times a day.

**Dr. Hommel's Hæmatogen. Hæmoglobin concentratum et Glycerinum purissimum.**—NICOLAY & Co., London.—This compound is essentially a purified and concentrated solution of hæmoglobin flavoured with pure glycerine and Malaga wine, in the proportion of four ounces of glycerine and two ounces of the wine in each pint.

Given in doses ranging from one to four teaspoonfuls it has a large range of utility in cases associated with anæmia and defective blood formation. We cannot have too many hæmatinics: the inorganic preparations of iron are usually the most rapid in action, and give therefore the best results; but there are many patients who fail to derive that benefit which we expect, and in many of them the albuminates of iron do better. The solution when much diluted shows the characteristic spectrum of hæmoglobin, and it also gives an abundant precipitate with picric acid.

---

**Tabloids.** — BURROUGHS, WELLCOME & Co., London. — **Quinine Hydrobromide**, gr. iij.— gr. v.—It is now generally admitted that the hydrobromic salt of quinine has distinct advantages over the combination with other acids, hence these tabloids should be especially useful for persons who say that they can never take quinine, and for cases where full doses are required.

**Codeine,** gr. ¼—gr. ½.—For diabetic patients these tabloids should be especially useful.

**Urotropine,** gr. v.—This drug appears to be rapidly establishing a reputation for itself as a diuretic and an antiseptic for the urinary tract, and the tabloid is a very convenient form of it.

**Blaud Pill, sugar-coated.**—Each tabloid contains eight grains of the Blaud compound in a very efficient and convenient form. This dose is likely to be of more service than the smaller ones which are generally given, and which often fail when larger doses succeed.

**Bismuth, Rhubarb and Soda.**—Contains bismuth subnitrate, gr. iij.; powdered rhubarb, gr. j.; sodium bicarbonate, gr. ij. It disintegrates readily, and should be a very useful combination.

**Chinosol** (Potassium oxy-quinolin sulphate) gr. v.—This drug has been in use for surgical purposes, and the soloid has been a convenient means of preparation of a solution, which does not coagulate albumen or injure instruments. Recently it has been given in quantities of fifteen to thirty grains daily in cases of tubercular diseases of the glands and bones, also in leprous ulcerations, and in dysentery.

---

**Cerebos Salt.** — CEREBOS LIMITED, Newcastle-on-Tyne.— Attention is being again directed to the frequency of underfeeding in phosphates, and the facility with which the deficiency may be remedied by the use of Cerebos salt, which contains a small but definite proportion of the mixed phosphates as found in wheaten bran.

---

**"Enule" Brand Rectal Suppositories.**—BURROUGHS, WELLCOME & Co., London.—These suppositories must facilitate rectal medication. A series of accurately dosed suppositories, of improved shape, and with each enule enclosed in a hermetically sealed sheath of tin-foil, is ready for use, and may be relied upon for promptness of action and therapeutic value.

Professor Caspari, in his *Treatise on Pharmacy*, page 390, says: "The usual shape of rectal suppositories is that of a cone with a rounded apex, but the difficulties of readily introducing these into the rectum, on account of the resistance offered by contraction of the sphincter muscle, has led to the designing of a *new shape*, by H. S. Wellcome, of London, the great advantage of which will become apparent when it is remembered that the bulbous end is inserted into the rectum first, and that as soon as the greatest diameter has been passed, expulsion of the suppository is impossible by reason of the very contractile force of the sphincter, which renders retention of the ordinary conical shape often so difficult."

The opium extract enule contains one grain in each. The sheath protects the contents from climatic and atmospheric influences.

The milk enule contains 10 grains (0.648 gm.) of concentrated peptone from milk, the caseine having been predigested.

The meat enule contains 8½ grains (0.551 gm.) of concentrated peptone from beef.

With these enules absorption may commence immediately after insertion, as it is not hindered by any coating or capsule of foreign matter. The protecting metallic sheath is of course to be removed before the insertion of the contents.

---

**Oxo Fluid Beef.**—G. STREET & Co., London.—This fluid is a very viscid condensed product made by the Liebig Company. One teaspoonful in a teacup of boiling water makes a palatable and stimulating draught. It is the outcome of many years experiments, and the promoters believe it to be the best fluid beef ever produced. They "do not hesitate to say that in composition, flavour, and potency it is better than any other fluid beef in the market." When much diluted it gives an abundant sediment, consisting for the most part of fragments of muscular fibres, and the fluid part shows the presence of an appreciable amount of albumen.

---

**Protene.**—THE PROTENE COMPANY, London.—On a former occasion (vol. xvii., 1899, p. 280) we directed attention to this milk product; and we have now again to allude to the great variety of food products of which Protene is the basis:

| Ref. No. | NAME OF PRODUCT. | PERCENTAGE COMPOSITION. | | | | |
|---|---|---|---|---|---|---|
| | | Nitrogenous Matter. (Proteids.) | Carbohydrates. (Starch, Sugar.) | Mineral Salts. | Non-nitrogenous Matter.* (Fats, &c.) | Moisture. |
| 3 | Protene Wheat Flour ... | 25 | 60 | 3 | 1 | 11 |
| 6 | Protene Household Bread (White) | 25 | 43 | 2 | 0 | 30 |
| 6 | ,, ,, (Brown) | 25 | 30 | 3 | 0 | 42 |
| 7 | **Protene Diabetic Bread ...** | **65** | **a trace** | **4** | **15** | **16** |
| 8 | **Protene Almond Bread ...** | **60** | **0** | **4** | **11** | **25** |
| 9 | Protene Gingerbread ... | 25 | 54 | 2 | 5 | 14 |
| 10 | **Protene Diabetic Gingerbread ... ... ...** | **40** | **18** | **2** | **29** | **11** |
| 11 | **Pure Protene Biscuits ...** | **60** | **a trace** | **4** | **28** | **8** |
| 12 | **Protene Medical Biscuits ...** | **50** | **10** | **3** | **29** | **8** |
| 13 | Protene Training Biscuits... | 50 | 20 | 2 | 22 | 6 |
| 14 | Protene Luncheon Biscuits | 40 | 26 | 2 | 21 | 11 |
| 15 | Protene Oatmeal Biscuits... | 45 | 25 | 2 | 23 | 5 |
| 18 | **Protene Fancy Biscuits (Diabetic)** | **40** | **16** | **2** | **36** | **6** |
| 19 | Protene Victoria Biscuits... | 30 | 37 | 2 | 20 | 11 |
| 21 | Protene Sponge Cakes ... | 25 | 56 | 1 | 0 | 18 |
| 23 | Protene Salt Sticks ... | 35 | 40 | 3 | 18 | 4 |
| 24 | Protene Rusks ... ... | 30 | 42 | 2 | 18 | 8 |
| 25 | **Protene Diabetic Rusks ...** | **50** | **16** | **2** | **27** | **5** |
| 26 | Protene Sponge Rusks .. | 25 | 59 | 1 | 0 | 15 |

Nos. 7, 8, 10, 11, 12, 18, 25, are especially prepared for diabetic patients, and should be of great service to them.

---

**Earl's I. and I. Food.**—The Infant and Invalid Food Co., London.—Of the making of foods there is no end, and it is difficult to choose among so many: nevertheless, a variety is often necessary, and it is sometimes convenient to resort to something out of the usual beaten track. The inventors of the "I. and I. Food" observing that certain cereals, although indigestible given separately, yet by combination are most easy of digestion, claim to "have successfully solved the problem which has hitherto baffled all previous attempts, by a combination of the most nutritive ingredients so scientifically and peculiarly proportioned, and cooked by a special process, that one facilitates the digestion of the other in a manner which renders it acceptable to the weakest infant." The following is the analysis of "I. and I. Food":—

| | |
|---|---|
| Moisture ... ... ... ... ... .. ... | 8.03 |
| Oil and Fat ... ... ... ... ... ... | 3.55 |
| Albuminoids ... ... ... ... ... ... | 13.19 |
| Digestible Carbohydrates ... ... ... ... | 72·03 |
| Fibre ... ... ... ... ... ... ... | 1.00 |
| Mineral Matter (Ash) ... ... ... ... ... | 2.20 |
| | 100.0 |

* This non-nitrogenous matter is not convertible into sugar.

It is not a predigested food; the combination of the various classes of food elements is well devised, and should be suitable for the maintenance of health or restoration to health. Where a cereal food is considered to be more suitable than milk, this preparation will provide it in a form which has been found to be eminently efficient.

**Cognet's Hemoneurol.**—A. Cognet, Paris; Roberts & Co., London.—A combination in granular form of oxyhemoglobine, kolanine, and glycero-phosphate of lime. The combination is one which naturally commends itself as a stimulant to and a regenerator of the blood and the tissues. Cognet's dragées and Cognet's capsules have long been well known and appreciated by those who like luxurious medication. The hemoneurol preparation is a novel and attractive one: it is pleasing to the taste, and will be acceptable to the most fastidious of patients. It is readily soluble in water, wine, tea, or any other beverage, and may be given in doses of three to four teaspoonfuls in the day.

